# Role of the Renin–Angiotensin–Aldosterone and Kinin–Kallikrein Systems in the Cardiovascular Complications of COVID-19 and Long COVID

**DOI:** 10.3390/ijms22158255

**Published:** 2021-07-31

**Authors:** Samantha L. Cooper, Eleanor Boyle, Sophie R. Jefferson, Calum R. A. Heslop, Pirathini Mohan, Gearry G. J. Mohanraj, Hamza A. Sidow, Rory C. P. Tan, Stephen J. Hill, Jeanette Woolard

**Affiliations:** 1 Division of Physiology, Pharmacology and Neuroscience, School of Life Sciences, University of Nottingham, Nottingham NG7 2UH, UK; Steve.Hill@nottingham.ac.uk; 2 Centre of Membrane Proteins and Receptors (COMPARE), School of Life Sciences, University of Nottingham, Nottingham NG7 2UH, UK; 3 School of Medicine, Queen’s Medical Centre, University of Nottingham, Nottingham NG7 2UH, UK; mzyeb10@nottingham.ac.uk (E.B.); mzysrj@nottingham.ac.uk (S.R.J.); mzycrh@nottingham.ac.uk (C.R.A.H.); mzypm14@nottingham.ac.uk (P.M.); mzyggm@nottingham.ac.uk (G.G.J.M.); mzyhasi@nottingham.ac.uk (H.A.S.); mzyrct@nottingham.ac.uk (R.C.P.T.)

**Keywords:** COVID-19, renin–angiotensin–aldosterone system, kinin–kallikrein system, cardiovascular system, long COVID

## Abstract

Severe Acute Respiratory Syndrome Coronavirus 2 (SARS-CoV-2) is the virus responsible for the COVID-19 pandemic. Patients may present as asymptomatic or demonstrate mild to severe and life-threatening symptoms. Although COVID-19 has a respiratory focus, there are major cardiovascular complications (CVCs) associated with infection. The reported CVCs include myocarditis, heart failure, arrhythmias, thromboembolism and blood pressure abnormalities. These occur, in part, because of dysregulation of the Renin–Angiotensin–Aldosterone System (RAAS) and Kinin–Kallikrein System (KKS). A major route by which SARS-CoV-2 gains cellular entry is via the docking of the viral spike (S) protein to the membrane-bound angiotensin converting enzyme 2 (ACE2). The roles of ACE2 within the cardiovascular and immune systems are vital to ensure homeostasis. The key routes for the development of CVCs and the recently described long COVID have been hypothesised as the direct consequences of the viral S protein/ACE2 axis, downregulation of ACE2 and the resulting damage inflicted by the immune response. Here, we review the impact of COVID-19 on the cardiovascular system, the mechanisms by which dysregulation of the RAAS and KKS can occur following virus infection and the future implications for pharmacological therapies.

## 1. Introduction

A novel coronavirus, termed severe acute respiratory syndrome coronavirus 2 (SARS-CoV-2), was first identified at the end of 2019 [1]. SARS-CoV-2 is responsible for the coronavirus disease 2019 (COVID-19) pandemic [1]. An array of clinical manifestations that vary in severity, from asymptomatic to acute respiratory distress syndrome (ARDS) and total organ failure are associated with COVID-19 [2,3]. Presently, over 180 million people worldwide have contracted COVID-19 and more than 3.9 million have died as a result of infection with SARS-CoV-2 [4].

Populations most at risk of hospitalisation have been found to be the elderly and those suffering from comorbidities such as cardiovascular disease and hypertension [5,6]. In many cases, underlying cardiovascular comorbidities were potentiated by SARS-CoV-2 infection and the resulting cardiovascular complications (CVCs) produced a greater mortality [7]. However, adverse cardiovascular events, such as arrhythmia were also reported in individuals who were young and did not have existing cardiovascular disease [8]. This suggests that the COVID-19 CVCs may result from the impact of the infection on the cardiovascular system independently of underlying conditions. Furthermore, an increasing number of recovered individuals report a multitude of symptoms that persist for months after the initial infection has cleared [9]. This has been termed “long COVID”. Strikingly, approximately 70% of a young, low risk population who experienced long COVID were found to have impairment of one or more organs [10]. Therefore, it has been hypothesised that direct and indirect SARS-CoV-2 mediated organ damage drives long COVID [10].

Due to the ubiquitous nature of long COVID and CVCs, it is important to identify the mechanisms underlying their occurrence in order to implement strategies to lessen the impact on overall health and future risk of chronic diseases. It is also crucial to consider the pathogenesis of SARS-CoV-2 and identify the homeostatic systems that are disrupted during progression of infection. Predominantly, the route of SARS-CoV-2 cellular entry has been considered to be key to this. A major route by which SARS-CoV-2 and the related coronavirus SARS-CoV-1 gain cellular entry is via the docking of the viral spike (S) protein to the membrane bound angiotensin converting enzyme 2 (ACE2) [11,12,13,14]. A number of scaffold proteins that facilitate virus internalisation alongside ACE2, such as neuropilin-1 and transmembrane protease serine 2 (TMPRSS2) have also been described [15]. Concomitantly, the roles of ACE2 within the cardiovascular and immune systems are vital to ensure homeostasis due to its involvement in the Renin–Angiotensin–Aldosterone System (RAAS) and the Kinin–Kallikrein System (KKS) [16]. The key routes for the development of CVCs and the recently described long COVID have been hypothesised as the direct consequences of the viral S protein/ACE2 axis and the resulting damage inflicted by the immune response. In particular, cytokine and bradykinin storms have been implicated in the worsened outcomes relating to COVID-19 [17,18].

A better understanding of the biological features of SARS-CoV-2 infection relevant to the cardiovascular system should enable us to delve deeper into the mechanisms responsible for CVCs and long COVID. This will be particularly important for identifying the risk of mortality in patients and identifying those at risk of developing long-term cardiovascular implications. Here, we have reviewed the cardiovascular consequences of SARS-CoV-2-induced ACE2 downregulation for the development of CVC and long COVID. The role of the RAAS in cardiovascular homeostasis has been extensively reviewed (see for example [19,20,21]) and, as a consequence, our focus is very much on the specific role of ACE2 in both the RAAS and KKS where it has a key role in regulating the metabolism of both angiotensin II (Ang II) and bradykinin (BK). As a consequence, we have also considered the implications of cytokine and bradykinin storms, as well as the co-morbidities that can lead to severe SARS-CoV-2 infections and CVCs. Finally, we consider the therapeutic potential of targets found within these pathological mechanisms. These discoveries have the potential to lead to pharmacological advancements that may result in the development of treatment/management strategies that could reduce COVID-19-related fatality rates. This will be particularly important in cases where patients are unable to receive vaccines or vaccination is ineffective [22].

## 2. The Role of ACE2 in the RAAS and KKS

The importance of ACE2 in the pathogenesis of SARS-CoV-2 infection and COVID-19 has been of particular interest in recent months [11]. The location of ACE2 within the body is thought to be key to determining the progression of disease by viruses that target this receptor [23]. Measurements of ACE2 mRNA levels have revealed high expression in the intestines, kidneys, heart and adipose tissues [24,25], with moderate and low levels discovered in the lungs and blood vessels, respectively [26]. If ACE2 endocytosis is the mechanism by which SARS-CoV-2 infiltrates host cells then by definition, high viral loads will lead to a reduction in ACE2 on the surface of cells [27,28]. There is strong evidence to support SARS-CoV-1 downregulation of ACE2 and/or shedding of ACE2 from the cell surface [12,13,29,30]. Given the fact that SARS-CoV-1 and SARS-CoV-2 both rely on the binding of the viral S glycoprotein to ACE2 for viral entry into cells it was therefore highly likely that ACE2 would also be downregulated by SARS-CoV-2 in COVID-19. This has now been confirmed by Lei et al. (2021) [31], Pedrosa et al. (2021) [32] and Sui et al. (2021) [33]. For example, Pedrosa et al. (2021) showed that the S protein can cause a 50% reduction in the 120KDa membrane-bound form of ACE2 in human alveolar type 2 A549 cells. This was also accompanied by a significant increase in the levels of soluble ACE2 (105 KDa) which is likely to be a consequence of ADAM17 [32]. A very large increase in the internalisation of both ACE2-GFP and SARS-CoV-1 S protein RBD-Fc was also observed in A549 cells on addition of the S protein [32]. These data, taken together, suggest that decreased levels of membrane-bound ACE2 may have a major role in the development of extra-pulmonary symptoms of COVID-19 and long COVID. Further, this reduction in ACE2 cell surface expression can cause a down regulation of the homeostatic roles of ACE2 in healthy individuals and may be responsible for the plethora of symptoms observed in COVID-19 [27].

ACE2 has many roles in normal physiology, particularly in terms of counter-regulation of the RAAS. Vital for long-term homeostatic maintenance of blood pressure (BP), fluid and electrolyte balance and cardiac function, the RAAS has been implicated in the disease progression of numerous viral infections and cardiovascular diseases [34,35,36,37].

**Figure 1:**
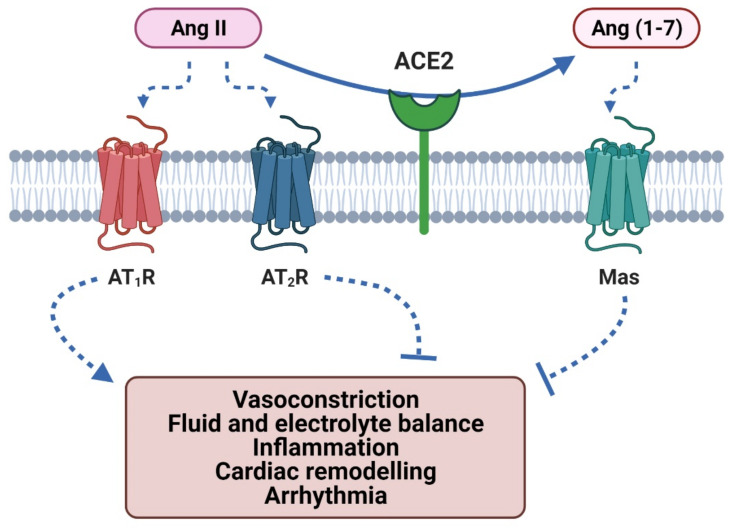
Effects of Ang II and its metabolite Ang (1-7). Ang II facilitates the “vasoconstrictive arm” of the RAAS via activation of angiotensin II type 1 receptors (AT_1_R). The “vasorelaxant arm” counteracts these effects via angiotensin II type 2 receptors (AT_2_Rs) and Mas receptors. Headed arrows show activation; flat-headed lines represent inhibition (created with Biorender.com).

**Figure 2:**
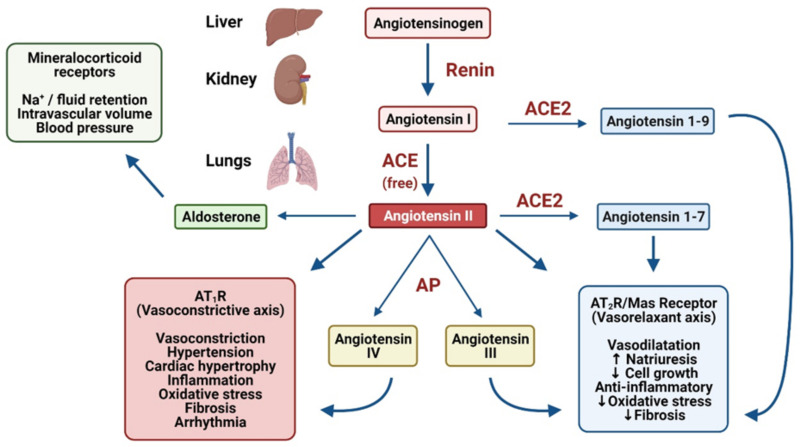
A summary of the action of enzymes involved in the RAAS. ACE, Angiotensin converting enzyme (ACE); AP, aminopeptidases. Figure created with Biorender.com.

In normal physiology the RAAS is tightly regulated with appropriate feedback loops and mechanisms to regulate the expression of key components. Nevertheless, the RAAS can be upregulated under pathological circumstances leading to dysregulation of inflammation, cell proliferation, apoptosis, angiogenesis and other cardiovascular responses (Figure 1 and Figure 2) [19].

As outlined in Figure 1, the RAAS is comprised of receptor signalling components that may enhance vasoconstriction and cardiac fibrosis (e.g., via Ang II type 1 receptor (AT_1_R) activation) or inhibit this change in vasoactive tone and provide cardio-protection (via activation of Ang II Type 2 receptors (AT_2_Rs) and Mas receptors) [38,39].

It is therefore unsurprising that the ‘AngII/AT_1_R axis’ has been implicated in a number of diseases and consequently there are many clinically available therapies that block signalling via AT_1_Rs [40]. Conversely, activation of AT_2_R by Ang II has been reported to produce opposing actions that attenuate the ‘AngII/AT_1_R axis’. In healthy individuals, these two components of the RAAS work synergistically to maintain homeostasis. However, RAAS dysregulation is associated with obesity, cardiovascular disease, hypertension and diabetes, all of which produce an elevated risk of mortality in COVID-19 patients [5,6]. Therefore, it is important to understand the regulation of the RAAS, as it could be key to determining mechanisms for SARS-CoV-2 mediated CVCs, severe COVID-19 and long COVID [5,6].

### 2.1. Angiotensin II Type-1 Receptor Activation

Activation of the RAAS involves multiple enzymatic reactions that synthesise and degrade angiotensin peptides, derived from angiotensinogen (Figure 2). The first step in the cascade is initiated by the release of renin from renal juxtaglomerular cells [40]. Renin release is triggered by sympathetic activation of β_1_ adrenoceptors, reduced renal perfusion pressure, and/or a decrease in blood sodium/chloride ion content [41,42].

Once in the circulation, this enzyme exerts local actions in a variety of tissues, including the liver, where angiotensinogen is hydrolysed by renin to angiotensin I (Ang I) [40]. The biological role of Ang I is not fully understood, although it is the known precursor to Ang II, which has been characterised extensively. A further enzyme, angiotensin converting enzyme (ACE), which is found in high concentrations in lung vascular endothelium, catalyses the conversion of Ang I to Ang II [43]. Ang II facilitates the physiological actions of the RAAS via interactions with the Ang II receptor subtypes, AT_1_R and AT_2_R, both of which are G protein-coupled receptors (GPCRs). When bound to AT_1_Rs, Ang II causes constriction of arterioles, elevated BP, facilitates cardiac hypertrophy and an increase in pulse rate (Figure 2) [40]. ACE2 cleaves Ang I and Ang II to form Ang(1–9) and Ang(1–7), respectively. Ang(1–7) induces vasodilatation, anti-inflammatory, and antifibrotic mediators via AT_2_R and Mas Receptors. Aminopeptidases (AP) convert Ang II into Ang III and IV which continue to exert cardiovascular and inflammatory effects [44].

The Ang II/AT_1_R axis is also implicated in oxidative stress which stimulates endothelial dysfunction, vessel inflammation, thrombosis, cardiac remodelling and insulin resistance [45,46]. In critically ill COVID-19 patients, Ang II levels are elevated and since the Ang II/AT_1_R axis is linked to cardiovascular dysregulation, this could contribute to COVID-19 CVCs [47]. For example, upregulated AT_1_R expression has been shown in patients with cardiovascular dysregulation, with notable links to arrhythmias and cardiac remodelling [48]. A global survey has demonstrated the association of COVID-19 and the development of arrhythmias in hospitalised patients, which is linked to high morbidity and mortality [49]. Although the development of arrhythmias is multifactorial and often accompanied by myocardial injury, cardiac remodelling, co-morbidities and pro-arrhythmic inflammation in COVID-19 [50], evidence suggests that Ang II and AT_1_R could be implicated in the development of these CVC through their involvement in calcium signalling (Table 1) [51,52].

The distinct pathophysiological features of AT_1_Rs are a result of their specific G-protein coupling and their differential expression levels in certain cell types [39,53] (Table 1).

Primarily in vascular smooth muscle cells (VSMCs) and in renal proximal tubules (RPTs), Ang II facilitates G_αq_ coupling via AT_1_Rs. This induces VSMC contractions and vessel constriction, which increases BP; this is associated with enhanced sodium water retention via G_αq_ coupling in RPTs (Table 1) [38,39]. Additionally, G_α12/13_ -mediated oxidative stress in cardiac fibroblasts and vascular endothelial cells has been implicated in cardiovascular disease and heart failure [60,61,62]. This highlights the significant role of AT_1_R activation of G_αq_ and G_α12/13_ signalling in the progression of cardiovascular and kidney dysfunction.

Although key to the vasoconstrictive arm of the RAAS, AT_1_Rs also activate protective mechanisms by signalling via G_αi2/3_, and receptor desensitisation is achieved via the recruitment of βarrestin2 [53]. For example, G_αi_ and βarrestin2 attenuate G_αq_ and G_α12/13_ signalling and downregulate the negative consequences of Ang II. The coupling of the various G protein subtypes facilitates distinct physiological responses that could be modulated by pharmacological intervention. Therefore, the suitability for AT_1_Rs as targets for COVID-19 CVCs should be investigated further.

Another component of the AngII/AT_1_R axis involves the steroid hormone aldosterone. Following stimulation of AT_1_Rs by Ang II and G_αq_ coupling, aldosterone synthesis is stimulated in the zona glomerulosa of the adrenal cortex [63,64]. Aldosterone secretion is also initiated by circulating potassium and adrenocorticotropic hormone levels [63]. Similar to Ang II, excess aldosterone has been implicated in hypertension and CVCs due to its regulatory role on cardiovascular and kidney function (Figure 2) [65]. In particular, through the activation of mineralocorticoid receptors, aldosterone modulates intravascular volume and BP through sodium retention in the kidney [66]. Therefore, it has been suggested that pharmacological modulation of AT_1_R and aldosterone signalling pathways may attenuate the deleterious effects of Ang II in cardiovascular diseases and COVID-19 CVCs [64].

### 2.2. Angiotensin II Type-2 Receptor Activation

In contrast to the vascular and cardiac effects described above, AT_2_Rs primarily generate opposing actions to the Ang II/AT_1_R axis, including vasodilatation, reduced BP, decreased platelet aggregation, increased insulin sensitivity and cardio-protection [67]. In normal physiology, the counter-regulatory role of AT_2_Rs on the Ang II/AT_1_R axis can prevent the development of cardiovascular dysfunction.

AT_2_R signalling is not well characterised, however, the opposing actions of AT_2_Rs may be a result of their differential G protein-coupling (Table 1). AT_2_Rs have been shown to signal via G_αs_ which activates mechanisms that result in cardiac regeneration, vasorelaxation, paracrine signalling, and protection from cardiac fibrosis, through bradykinin/cGMP/NO production (Table 1) [68]. There is also evidence of G_αi_ coupling [54]. However, studies investigating the definitive role of G_αi_ coupling in AT_2_R signalling remain inconclusive. Contrastingly, several studies suggest a non-canonical signalling route that bypasses G protein and β-arrestin pathways to directly activate ERK1/2 and NOS [55,56]. As such, further work is required to elucidate the exact mechanisms responsible for AT_2_R physiological responses. Utilising new technologies such as the G protein and signalling biosensors described by Namkung et al., for the AT_1_R may be key to improving our understanding of AT_2_R function [53]. This could culminate in the identification of ligands that stimulate AT_2_Rs to reduce BP and protect against Ang II mediated cardiovascular dysfunction [69]. It should be noted however, that in a diseased state AT_2_Rs have been shown to amplify bradykinin-mediated inflammation and there is conflicting evidence of the involvement of AT_2_Rs in left ventricular hypertrophy [58]. Therefore, it is important to understand AT_2_R signalling as over stimulation of AT_2_Rs, potentially by Ang II, could contribute to COVID-19 CVCs.

Another important component of the cardiovascular response is ACE2. The role of ACE2 within SARS-CoV-1 and SARS-CoV-2 pathogenesis has been highlighted, demonstrating similarities with the associated symptoms [11]. However, ACE2 is also important for normal RAAS regulation. In the RAAS, ACE2 downregulates Ang II by degrading Ang I into Ang(1-9) and degrading Ang II to Ang(1-7) (Figure 1 and Figure 2). Ang(1-9) mediates its actions via the AT_2_R and produces the protective responses such as anti-fibrotic and anti-inflammatory effects [70]. Moreover, Ang(1-7) has been reported to bind to another GPCR, the Mas receptor (MasR), which can exert NO-dependent vasorelaxation and protect against cardiac remodelling; Ang(1-7) also acts as a β-arrestin biased agonist at AT_1_Rs [71,72,73]. This induces AT_1_R desensitisation and internalisation, which attenuates the Ang II mediated cardiovascular dysfunction [57]. There is a report, however, that Ang(1-7) does not bind to MasR or elicit MasR-mediated signalling [74]. Instead, these authors provided evidence that Ang(1-7) was able to bind to AT_1_ and AT_2_ receptors (Ki values of 233nM and 288nM, respectively) and elicit potent inhibition of Ang II-stimulated inositol phosphate accumulation and ERK/2 activation in rat aortic endothelial cells [74]. In addition, as ACE2 cleaves Ang II and its precursor Ang I, it indirectly downregulates Ang II mediated pathophysiological effects. The cleaved metabolites then facilitate protection of endothelial function, prevention of thrombosis, inflammatory responses and cardiac remodelling via their respective receptors [16,72,75].

Similar to the AT_2_R, the MasR has not been extensively characterised and there are many conflicting theories surrounding its function. Initially, the MasR was postulated to stimulate the cardiovascular protective functions of Ang(1-7) [76]. However, more recent studies suggest that MasR is constitutively active and there is no observable G protein activation upon Ang(1-7) binding [71]. Interestingly, ACE2 and MasR are expressed in the same tissues, which could imply that together they co-ordinate tissue specific protection [58]. Clearly, the balance between Ang II, Ang(1-7) and Ang(1-9) dictates the physiological effects of RAAS, hence why modulation is central to cardiovascular disease management [16]. Targeting elements of the RAAS may also be key to correcting the cardiovascular dysfunction observed in COVID-19 patients.

## 3. Bradykinin Signalling: The Kinin–Kallikrein System

Considered an extension of the RAAS, the Kinin–Kallikrein system (KKS) also regulates BP [77]. The KKS mediates opposing actions to the RAAS by inducing arterial vasodilation [78]. The KKS is also involved in the regulation of tissue repair, inflammation, cell proliferation and platelet aggregation.

**Figure 3:**
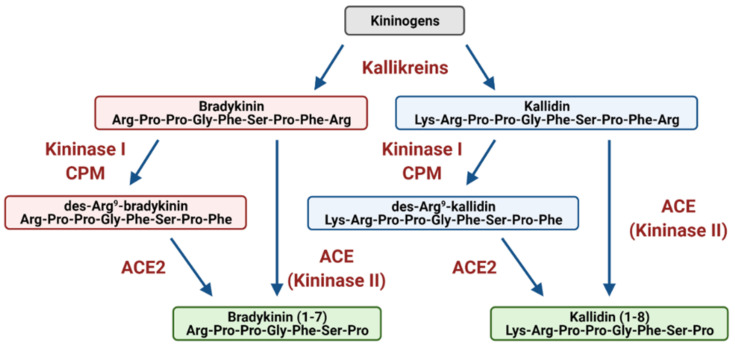
The Kallikrein–Kinin/Bradykinin System. Kallikrein serine proteases cleave kininogens to release the vasoactive peptides bradykinin (BK) and kallidin (KD). The peptidase Kininase I (carboxypeptidase M, CPM) further cleaves BK and KD into the active des-Arg^9^-bradykinin (DABK) and des-Arg^10^-kallidin (DAKD). Kininase II or ACE, inactivates the KKS by degrading BK, KD, DABK and DAKD into inactive metabolites. Similarly, ACE2 metabolizes DABK to BK (1-7) and DAKD to KD (1-8) [79] (created with Biorender.com).

The KKS is comprised of kallikreins serine proteases which cleave kininogens to release the vasoactive peptides bradykinin (BK) and kallidin (KD) (Figure 3). The peptidase Kininase I further cleaves BK and KD into the active metabolites des-Arg^9^-bradykinin (DABK) and des-Arg^10^-kallidin (DAKD) [79]. These kinins transmit their biological effects by activating the GPCRs, bradykinin-1 (B_1_) and bradykinin-2 (B_2_) receptors.

BK and KD bind to B_2_ receptors, while DABK and DAKD bind to B_1_ receptors. Both BK receptors mediate indirect cardio-protection, vasodilatation, coronary flow increase, reactive oxygen species (ROS) release and anti-thrombogenic effects [78]. To achieve these physiological effects, B_1_ and B_2_ receptors couple to G_αi_ and G_αq_ proteins. G_αi_ facilitates the release of locally acting vasodilators and inflammatory mediators such as, arachidonic acid and prostaglandin (Table 1), whereas G_αq_ protein coupling increases intracellular Ca^2+^ and NO-dependant vasorelaxation (Table 1) [78].

B_2_ receptors are ubiquitously expressed in all tissues. In contrast, B_1_ receptors are not usually expressed in normal physiology, and instead are upregulated during cellular stress and inflammation, particularly, in response to elevated COX-2 and iNOS levels [59]. There is evidence of cross talk between B_1_ and B_2_ receptors, as continuous stimulation of B_2_ receptors results in upregulation of B_1_ receptors. Additionally, B_1_ receptors do not internalise or desensitise; therefore, their stimulation can induce sustained Ca^2+^ elevations and long-term inflammation and inflammatory pain [59]. As such, excessive stimulation of B_1_ receptors has been implicated in hyper-inflammation and may be linked to the cytokine storm observed in severe cases of COVID-19 disease.

Fortunately, there are intrinsic regulatory components of the KKS that are linked to the RAAS. Firstly, located in endothelium, Kininase II, also known as ACE, inactivates the KKS by degrading BK and KD into inactive metabolites (Figure 3). This rapid degradation in local blood vessels causes the actions of the KKS to remain tissue specific. Secondly, in the lungs, ACE2 degrades DABK to BK (1-7) and therefore attenuates DABK mediated inflammation via B_1_ receptor activation [79,80]. The actions of ACE and ACE2 effectively down regulate the KKS and permit the return of homeostasis [81].

In a diseased state, such as hypertension, over activation or expression of ACE constitutes downregulation of the KKS and upregulation of the RAAS. This not only prevents the cardioprotective and antihypertensive actions of the KKS, but also results in over stimulation of RAAS, which potentiates cardiovascular adverse events and organ damage [82]. Many ACE Inhibitors (ACEi) have a higher affinity for the BK binding pocket of ACE [83]. This prevents the breakdown of BK and therefore the downregulation of the KKS by ACE. The accumulation of BK in upper and lower respiratory tracts sensitise sensory neurones that release inflammatory mediators, such as neurokinin A and substance P. This stimulates contraction of the smooth muscles within the airway, which is postulated to be a mechanism responsible for the dry cough often observed during ACEi treatment [84].

**Figure 4:**
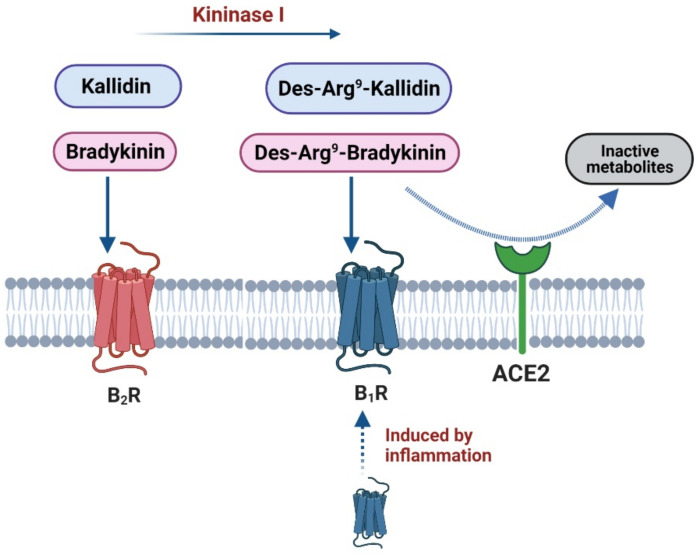
Receptor stimulation of B_1_ and B_2_ receptors. Kallidin (KD) and Bradykinin (BK) act principally on B_2_ receptors, while Des-Arg^9^-Kallidin and Des-Arg^9^-Bradykinin act on B_1_ receptors. As shown in the figure, the B_1_ receptor is induced by inflammation. ACE2 plays a role in the degradation of Des-Arg^9^-Kallidin and Des-Arg^9^-Bradykinin (created with BioRender.com).

Accumulation of BK or upregulation of KKS signalling has also been postulated as a mechanism causing the symptom of the COVID-19 dry cough. During SARS-CoV-2 infection, ACE2 activity is depleted as a result of virus internalisation mechanisms [27]. Loss of ACE2 function prevents DABK degradation and results in prolonged activation of B_1_ receptors (Figure 4). This enhances smooth muscle contraction, lung injury and inflammation, all of which contribute to pulmonary symptoms, including a dry cough [27,80]. It should also be noted that many other symptoms of COVID-19, including, fatigue, vomiting, diarrhoea and headaches are found in conditions where BK levels and vascular permeability are elevated, such as pulmonary angio-oedema [85,86]. It has been suggested that a combination of a cytokine and a BK storm may constitute severe COVID-19 symptoms and may be linked to the observed CVCs [77].

## 4. Cytokine Storm

Early reports and evaluation of clinical data suggested that a cytokine storm is associated with COVID-19 severity and may be a cause of increased mortality [18]. A cytokine storm has been described as a potentially fatal systemic inflammatory syndrome that involves accumulation of immune cells and hyper-inflammation, facilitated by cytokines and chemical mediators [18]. Clinical manifestations of the cytokine storm present as ARDS, hypoxaemia, hypotension, thrombosis, haemorrhages; and can induce renal failure, liver injury, encephalopathy and cardiomyopathy [18]. Transcriptomic and proteomic analysis of bronchoalveolar lavage fluid from COVID-19 patients confirmed robust chemokine, cytokine and interferon (IFN) responses, which were accompanied by neutrophil and monocyte-derived macrophage infiltration [87,88].

The mechanisms responsible for this cytokine storm have been proposed to initially arise following SARS-CoV-2 infection of respiratory epithelial cells and the release of viral nucleic acid, which provoked elevations in pro-inflammatory cytokines and IFN release by CD4^+^ T cells [89]. During viral infections, pattern recognition receptors (e.g., Toll-like receptors) can sense a variety of pathogen-associated molecular patterns displayed by viruses (e.g., envelope glycoproteins, single and double-stranded nucleic acids), which stimulate transcription of Interleukin-6 (IL-6) and other pro-inflammatory cytokines [90,91]. IL-6 and tumour necrosis factor α (TNFα) are implicated in the progression of the cytokine storm as they activate the cytokine producing pathway, Nuclear factor-κB (NF-κB), which recruit immune cells such as neutrophils, monocytes and macrophages [92,93].

This cytokine-mediated regulation of immune responses functions to protect tissues from infection related injury by modulating the release of chemokines, adhesion molecules and apoptotic regulators [94]. However, during a cytokine storm, these processes become dysregulated [18]. Subsequently, as the virus replicates and viral load increases, the immune cell recruitment, cytokine and chemokine release intensifies. The exponential growth of the resulting inflammation corresponds to the localised tissue and blood vessel damage [18]. The inflammation thereby cascades and amplifies the inflammatory response further. The damage inflicted on the lungs by hyper-inflammation causes hypotension and hypoxaemia, which in turn can contribute to hypoxia-mediated myocardial injury [95].

Approximately, 7–28% of COVID-19 patients were reported to have acute myocardial injury, defined by elevated troponin levels [3,96,97]. A proportion of these patients demonstrated evidence of SARS-CoV-2 mediated myocarditis, an acute inflammation of the myocardium [95]. This myocarditis caused an upregulation of inflammatory mediators, suggested to be associated with a cytokine storm. A combination of the cytokine storm and myocardial injury increases the metabolic rate and oxygen demand of the heart. This creates a supply and demand imbalance that intensifies myocardial load. The resulting hypoxia, metabolic acidosis and cardiac injury increases the risk of arrhythmias and cardiac arrest [95]. Meta-analysis of COVID-19 patients from 11 countries has shown that 20.3% of those who were hospitalised and developed arrhythmia resulted in fatality [98]. In other viral infections, consequences of inflammation-derived cardiac injury include dysfunctional repolarisation and action potential conduction [99]. These are a result of altered intracellular coupling, contribute to abnormal calcium ion handling and downregulation of K^+^ channels [51,52,99,100]. Myocardial injuries have also been observed in mild cases of COVID-19 and have been thought to contribute to long COVID occurrence [10]. Therefore, it is important to consider the impact of the cytokine storm on the cardiovascular effects of COVID-19 as all patients could be at risk of developing long COVID.

Recognition of the COVID-19 cytokine storm has led to the investigation of cytokine-directed therapies [101]. For example, the IL-6 monoclonal antibody, Tocilizumab, tested in phase II trials, has been shown to reduce COVID-19 lethality rate [102]. Although potentially effective, a caveat to this approach is that IL-6 and other cytokines are essential for a healthy response to both SARS-CoV-2 and other pathogens. Thus, inhibition of cytokine signalling could impair the clearance of secondary infections that may also result in poor outcomes [102]. It is also important to consider the appropriate time for administration as if given too early, cytokine directed therapies could limit COVID-19 recovery.

In addition to a virus driven cytokine storm, inflammatory mediators are indirectly upregulated in response to Ang II accumulation resulting from ACE2 downregulation by SARS-CoV-2 [27,103]. Activation of AT_1_Rs by Ang II can stimulate the NF-κB pathway, which amplifies TNFα and IL-6 release. Increased levels of circulating aldosterone have also been suggested to elevate IL-6 levels and this has been linked to lung injury in COVID-19 [104]. Therefore, it has been proposed that dysregulation of elements of the RAAS and KKS by SARS-CoV-2 could potentiate the cytokine storm or generate a bradykinin storm (see below) that may facilitate cardiovascular dysregulation and increase the risk of long COVID, irrespective of pre-existing cardiovascular disease.

## 5. Bradykinin Storm

Although a cytokine storm is often cited as the leading cause of the severe COVID-19 symptoms, recent findings suggest that BK and the KKS may play a more prominent role via a newly described bradykinin storm [17]. Downregulation of ACE2 by SARS-CoV-2 coupling and internalisation facilitates the depletion of ACE2′s catalytic products, such as Ang(1-7) and Ang(1-9) [103]. These catalytic products facilitate vasodilatation and have anti-inflammatory roles [17]. Perhaps unsurprisingly, through a reduction in these activities the bradykinin storm is associated with BP dysfunction and inflammation [17]. Additionally, there is an increase in the levels of ACE2 substrates, such as Ang II and DABK, which are pro-inflammatory mediators and could contribute to the acute lung damage observed in COVID-19 [17]. Therefore, the bradykinin storm could be result of the SARS-CoV-2 mediated reduction in ACE2 availability and the down regulation of DABK degradation [17]. The effect of increased levels of DABK will be further exacerbated by the increase in B_1_ receptor expression that results from cellular stress and inflammation [59]. An accumulation of BK has been detected in bronchoalveolar lavage fluid from COVID-19 patients and this has been linked to the symptoms associated with COVID-19 [77]. As the proteins described above are also major components of the RAAS and KKS, they have been linked to both the pathophysiology of SARS-CoV-2 and the corresponding CVCs.

**Figure 5:**
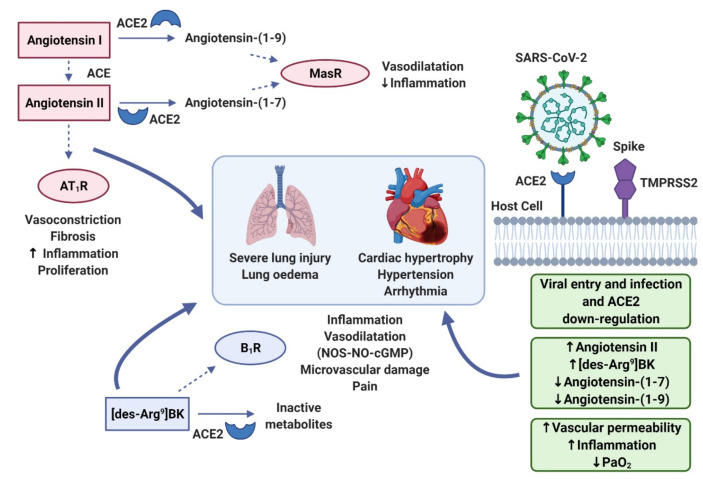
RAAS and Bradykinin Systems in SAR-CoV-2 infection. SARS-CoV-2 infection results in interaction with transmembrane protease, serine 2 (TMPRSS2) and ACE2, leading to subsequent down regulation of ACE2 [27]. This loss of ACE2 function leads to decreased metabolism of Ang II to Ang(1-7) and Ang(1-9). This leads to an increased activation of AT_1_R by Ang II and reduced activation of MasR. The resulting increased inflammation also leads to an increased expression of B_1_Rs. As a consequence, AT_1_R and B_1_ receptor stimulation facilitates the pathophysiological responses associated with COVID-19, such as ARDS and CVCs (Figure adapted from [107]; created with Biorender.com).

As detailed in Figure 5, within the RAAS, Ang II mediates some of the pathophysiological effects of SARS-CoV-2 through the binding to AT_1_Rs (Figure 5) [17,40]. These pathophysiological effects can increase AT_2_R expression. Additionally, AT_2_Rs are also highly expressed in the lungs in late adulthood and are upregulated in all areas in response to inflammation and tissue damage [105]. Although generally regarded as a counter-regulator to the pathophysiological effects of Ang II, AT_2_Rs also facilitate the upregulation of B_2_ receptor signalling [68]. Consequently, this induces vasodilatation, increased vascular permeability and inflammation in these tissue specific areas [54]. In muscles, the common symptom of myalgia could be linked to prostaglandin E2 release by B_2_ receptors [106].

Furthermore, accumulation of DABK promotes increased activation of B_1_ receptors (Figure 5). B_1_ receptors promote sustained inflammation and therefore contribute further to this BK receptor-mediated inflammation via the upregulation of pro-inflammatory cytokines such as IL-1 and IL-6 [59]. Notably, expression of B_1_ receptors is also upregulated in response to localised inflammation [59]. A major impetus for the development of the bradykinin storm hypothesis was the study of Garvin et al. (2020) [77] who undertook a differential gene expression analysis of RAAS genes in cells from bronchoalveolar lavage samples taken from severely affected COVID-19 patients. They found that expression (RNA-Seq) of all kallikreins and kininogens were upregulated and this was accompanied by a 2945-fold and a 207-fold increase in B_1_ and B_2_ receptors, respectively [77]. However, it is worth noting that this will be partly mediated by the infiltration of inflammatory cells into the lungs as a consequence of the ongoing COVID-19 infection. The work from Garvin and colleagues builds on a study that proposed that depletion of ACE2 in COVID-19 patients, and upregulation of B_1_/B_2_ receptors, might lead to vascular leakage in the lungs leading to angio-oedema [86]. Interestingly, the selective B_2_ receptor antagonist icatibant and the monoclonal antibody lanadelumab (which inhibits plasma kallikrein activity; [108]) are in clinical use for hereditary angio-oedema.

Studies have also shown that regulators of the KKS are down regulated in COVID-19. For example, in intensive care unit (ICU) COVID-19 patients there is evidence of a reduction in serpin family A member 12 (SERPINA12) and dipeptidyl peptidase 4 (DPPD-4), which are down regulated by IL-6 [109,110,111]. SERPINA12 and DPPD-4 are also tissue kallikrein and BK suppressors, subsequently, the down regulation of SERPINA12 corresponds to an increase in bradykinin activity [111]. This can result in the development of tissue specific angio-oedema which is a theorised mechanism for pulmonary angio-oedema [85] and the loss of taste and smell, a common symptom of COVID-19 [112].

In severe cases of COVID-19 infection, the initial over-stimulation of the BK receptors could potentiate inflammation that becomes progressively worse and leads to more severe symptoms and worse patient outcomes [80,111]. Increased vascular permeability allows neutrophil infiltration and the subsequent release of inflammatory mediators, including IL-6 and TNFα. In the lungs, this can lead to pulmonary angio-oedema which has deleterious effects on blood oxygen saturation levels [80,85]. In the heart and blood vessels, this bradykinin storm increases myocardial and endothelial dysfunction due to fibrotic accumulations; cardiac and vascular remodelling can also lead to severe cardiovascular symptoms that will be described in more detail later. Therefore, the bradykinin storm has been implicated in a decreased efficiency of the cardiovascular system. A culmination of all of these processes may be responsible for COVID-19 fatalities.

It is possible that in addition to a virus-mediated cytokine storm, dysregulation of the RAAS and KKS by SARS-CoV-2/ACE2 contribute to the hyper-inflammation observed in severe cases of COVID-19 and to the development of CVCs [113]. Indeed, the clinical manifestation of pulmonary angio-oedema in hospitalised COVID-19 patients could imply that bradykinin signalling has been upregulated to pathophysiological levels [86,111]. Therefore, the implications of a bradykinin storm and RAAS dysregulation could also delineate the cumulative risk of developing severe COVID-19 in elderly populations and in particular, those with cardiovascular comorbidities.

## 6. RAAS Involvement in Severe COVID-19 in Patients with Co-Morbidities

Severe COVID-19 is characterised by significant hypoxaemia associated with progressive respiratory failure [114]. Patients often require ventilation, and their risk of mortality is extremely high [114]. Throughout the pandemic, COVID-19 related fatalities have been linked to pre-existing health conditions [115,116]. Reports demonstrated that underlying conditions were present in 94% of COVID-19 deaths (CDC COVID-19 Response Team, 2020) [96]. The most common comorbidities of severe COVID-19 were hypertension (33–43%), diabetes (14–19%) and cardiovascular disease (8%) [96]. As these comorbidities are more prevalent in the aging population, they could be a factor in the higher fatality rates observed in the elderly [115]. It is important to investigate the implications of comorbidities in COVID-19 so that preventative care can be established to identify those patients most at risk of developing severe COVID-19 and reduce fatality rates in vulnerable populations.

### 6.1. Thromboembolism

Prior venous thromboembolism is reported to occur in approximately 7.5% of COVID-19 incidents and of those the mortality rate was over 1% [117]. Moreover, it has been suggested that COVID-19 may induce thrombotic and coagulation abnormalities that promote a hypercoagulable state [118]. In 25–43% of ICU patients, arterial and venous thromboembolic events were a severe CVC of COVID-19, demonstrating a strong link between the development of thrombotic complications and severe COVID-19 [119,120,121,122].

Endothelial injury has been the most theorised mechanisms for COVID-19 mediated aggravated thrombotic and coagulation abnormalities [123]. Several modalities of endothelial injury have been hypothesised and these include an imbalance of ACE2 regulation as a result of SARS-CoV-2 interactions; and inflammation mediated by pericyte dysfunction, activation of the complement system and a cytokine storm [123,124].

As mentioned previously, ACE2 is expressed throughout the cardiovascular system, particularly in vascular regulatory cells such as pericytes and endothelial cells [26]. Since ACE2 is the established route for SARS-CoV-2 cellular internalisation and host cell death typically occurs in most viral infections, COVID-19 can therefore result in a decline in vasculature regulatory cells, culminating in vascular injury and dysfunction [124]. Vascular endothelial injury has been shown to causes thrombocytopoenia and reduction of natural anticoagulants, in addition to thrombotic disseminated intravascular coagulopathy [124].

Furthermore, virus-mediated cellular death promotes the formation of the NOD-, LRR- and pyrin domain-containing protein 3 (NLRP3) inflammasome [125]. The NLRP3 inflammasome regulates the release of pro-inflammatory cytokines, which can lead to a systemic response [126]. The accumulation of inflammatory mediators (including IL-6 and IL-8) and the subsequent cytokine storm has been shown to lead to a hypercoagulable state via a reduction in fibrolysis and stimulation of the tissue factor pathway [127]. Tissue factor expression has been shown to be upregulated in macrophages, neutrophil extracellular traps (NETs) and platelets, and its activation has been linked to the upregulation of coagulation factors VII to VIIa which promote clot formation [128]. Activation of tissue factor has also been associated with the down regulation of ACE2 and the subsequent accumulation of Ang II, thus indicating links to the COVID-19 induced pro-thrombotic state and RAAS dysregulation [129].

It should also be noted that similar mechanisms to those detailed above have been considered as a cause for the recently reported thrombotic events associated with COVID-19 adenovirus expressing spike protein vaccinations [130]. Specifically, capture of adenovirus by heparin sulphate chains could activate a compliment mediated cytokine storm, resulting in coagulation cascades [131]. Alternatively, S protein expressed by adenovirus-infected endothelial cells and subsequent interactions with ACE2 could downregulate ACE2 and increase the risk of thrombotic events [131]. However, the exact mechanisms of vaccine related thrombotic events are yet to be fully characterised.

Fortunately, antithrombotic treatments, such as statins and antiplatelet therapies in high risk COVID-19 patients have demonstrated potential benefits and decreased the risk of both venous thromboembolism and mortality [117]. Alongside this, the monitoring and management of prior thrombotic events and RAAS dysregulation should also be considered in hospitalised COVID-19 patients and those receiving vaccines.

### 6.2. Hypertension

There is a clinically significant risk for hypertensive patients who contract COVID-19. During the peak of the initial outbreak in Italy, approximately 49% of COVID-19 related ICU admissions had hypertension, and of those 38% did not recover [132]. Several theories have been proposed for the positive correlation between hypertension and COVID-19 severity.

There is a strong consensus that ACE2 has a regulatory role in the development of hypertension and the severity of COVID-19. As described earlier, ACE2 downregulates the Ang II/AT_1_R pathway and BK signalling and therefore indirectly reduces the mediated pathophysiological effects of the RAAS and KKS [133,134,135]. The down regulation of ACE2 that accompanies the uptake of SARS-CoV-2 into the cell prevents the counter-regulatory role of ACE2. Concomitantly, many patients with hypertension already possess elevated Ang II levels [37,40]. This may be contributed to by phenotypic variations in ACE2 that predispose individuals to hypertension. For example, several ACE2 polymorphisms result in the downregulation of ACE2 [135]. Therefore, SARS-CoV-2 infection in these individuals could potentiate the pre-existing pathological levels of Ang II which leads to worsened symptoms of hypertension and increases the risk of stroke and heart failure [136].

Moreover, underlying hypertension is often accompanied by multiple morbidities such as obesity, diabetes and kidney disease, all of which contribute to reduced longevity in general and worse clinical outcomes of COVID-19 [137]. The poor outcomes can be partly attributed to the ability of COVID-19 to facilitate a bradykinin and cytokine storm. Underlying inflammation or aberrant immune responses associated with these conditions may be accelerated by this hyper-inflammation in multiple organs. The resulting organ damage increases the risk of total organ failure and mortality [136].

In addition, immune cell infiltration of blood vessels, kidneys, heart and nervous system can promote hypertension [138]. Therefore, the immune response generated by SARS-CoV-2 could potentially exacerbate the existing hypertension. Additionally, hypertension predisposes individuals to chronic kidney disease, coronary artery disease, stroke, left ventricular hypertrophy, and heart failure [139]. These conditions are primarily observed in elderly hypertensive patients; therefore, the additional risks of hypertension and COVID-19 can be life limiting. This could explain why elderly hypertensive patients had the highest mortality rates during the COVID-19 pandemic [47,140,141].

Fortunately, patients with controlled hypertension have shown improved outcomes following SARS-CoV-2 infection in comparison to those with uncontrolled hypertension [142]. In many cases, the use of ACEi and angiotensin receptor blockers (ARBs) effectively re-establish RAAS homeostasis by reducing Ang II pathophysiological signalling and unsurprisingly, these mechanisms may reduce the progression of severe COVID-19 [40].

However, a confounding argument for the use of ACEi and ARBs during the pandemic has generated much controversy. These hypotheses suggested that ACEi and ARBs could potentiate SARS-CoV-2 infection. This argument was based on evidence of ACEi and ARBs increasing ACE2 expression [32,142]. It has been suggested that an abundance of alveolar ACE2 might facilitate increased SARS-CoV-2 cellular entry and viral replication. This would increase viral load and therefore COVID-19 severity [142,143]. In contrast to this, studies have shown that high levels of ACE2 expressing cells, particularly in children, can mediate protective effects against COVID-19 [28]. Interestingly, the study by Pedrosa et al. (2021) also showed that treatment with candesartan or captopril could prevent the depletion of ACE2 induced by S protein [32]. Withdrawal of ACEi and ARB treatment can result in a complete reversal of antihypertensive and cardio-protective effects in patients with heart failure and reduced ejection fraction [142,144] and so withdrawal of RAAS is clearly not recommended. A recent meta-analysis in animal models of human disease confirmed that ACE2 overexpression as a consequence of inhibition of the RAAS was rare [145]. This finding was confirmed in a meta-analysis of the risk of mortality in hospitalised patients with COVID-19 [146].

### 6.3. Cardiovascular Disease

The third comorbidity that is closely related to the fatal outcomes of COVID-19 is cardiovascular disease [147]. Previous studies demonstrated that patients with cardiovascular diseases were both more susceptible to SARS-CoV-2 infection and had an increased risk of developing severe COVID-19 [148].

It has been suggested that increased susceptibility to SARS-CoV-2 infection in cardiovascular diseases is associated with ACE2 expression. Following myocardial infarction and heart failure, ACE2 expression is upregulated in cells including macrophages, endothelial cells, smooth muscle cells, and cardiomyocytes [143]. As discussed above, elevated ACE2 levels may present increased opportunities for SARS-CoV-2 to gain cellular entry [149]. Sequentially, this increases the level of viral replication and subsequently, viral load, which has been implicated in COVID-19 severity [143,149].

Conversely, polymorphisms that downregulate ACE2 have also been identified in cardiovascular disease [135]. Theoretically, this reduced ACE2 activity could be potentiated by the indirect down regulation of ACE2 by SARS-CoV-2 cellular internalisation and this could impair clearance of Ang II as ACE2 is unable to metabolise Ang II [136]. The resulting accumulation of Ang II could further exacerbate impaired cardiac function, arrhythmia and hypertrophy [24,150,151,152].

In addition, dysregulated KKS may potentiate cardiovascular diseases. For example, Ang II upregulation in cardiovascular disease has been associated with DABK-B_1_ receptor mediated cardiac hypertrophy [153]. Additionally, excess BK has been implicated in hypokalaemia, which can lead to arrhythmia and sudden cardiac death [36,154]. Hyper-inflammation of the myocardium by the cytokine and bradykinin storms can further contribute to pre-existing myocardial injury. As such, the pathogenic impact of SARS-CoV-2 on ACE2, Ang II, BK and DABK in cardiovascular disease may be a mechanistic pathway for worsened outcomes of COVID-19 [86].

### 6.4. Diabetes

Diabetes is another highly prevalent comorbidity of COVID-19 that has been reported as the strongest predictor of mortality out of the most common co-morbidities [155,156]. Specifically, patients with poorly controlled blood glucose levels (determined by glycated haemoglobin) have an increased risk of developing severe COVID-19 symptoms [157,158]. Alarmingly, cases of new onset diabetes or diabetic ketoacidosis have also been reported in patients [159]. This can be explained by the recent data from COVID-19 patient samples indicating SARS-CoV-2-mediated inflammation of the pancreas accompanied by acute damage to B cells and reduced expression of insulin [160]. It has been suggested that the level of ACE2 expression in the pancreas also correlates with the extent of pancreatic damage following SARS-CoV-2 infection [161]. A further explanation for the SARS-CoV-2 mediated pancreatic damage could be the amplification of a cytokine storm by the hyperglycaemic environment, since elevated inflammatory cytokine levels were detected in patient samples [160].

Patients with diabetes chronically suffer from disproportionate hyperglycaemic responses, which can lead to a hindered immune system [162] and the development of both micro- and macro-vascular complications [163]. These include retinopathy, neuropathy and nephropathy and ischaemic heart disease, peripheral vascular disease, and cerebrovascular disease. Subsequently, diabetic patients have a significant risk of developing COVID-19 related CVCs such as acute myocarditis, acute heart failure, acute myocardial infarction and new on-set atrial fibrillation, compared to non-diabetics [164].

## 7. Long COVID

The evolving critical care treatments of severe COVID-19 have drastically reduced mortality rates in comparison to the start of the pandemic [165]. Additionally, the successful roll-out of the various COVID-19 vaccines has seen declines in COVID-19 related hospital admissions [22]. However, there is still a high prevalence of recovered individuals reporting ongoing symptoms or long COVID [166]. While the majority of people recover within approximately 2 weeks, recent findings show that around one in ten people suffer with multi-organ symptoms, including the cardiovascular system, and complications that persist for more than 12 weeks, post the initial onset of acute infection [9]. The list of long COVID symptoms includes dyspnoea, chronic cough and extreme fatigue. With particular relevance to this review, long COVID has been associated with numerous CVCs such as myocarditis, microvascular angina, cardiac arrhythmias and BP abnormalities [166].

Strikingly, there are large variations in the estimated prevalence of long COVID in different populations and COVID-19 severities [167]. Recent retrospective large cohort data have shown that approximately 29.4% of recovered COVID-19 patients were readmitted to hospital and 12.3% died following discharge as a consequence of COVID-19 related adverse events [167]. Many studies have predicted that recovered COVID-19 ICU patients will endure similar long-lasting symptoms that may remain for years to come [167,168]. Further evidence suggests that patients are at risk of developing serious chronic conditions in the future, as indicated by post COVID-19 diagnoses of major adverse cardiovascular events, including chronic kidney disease, chronic liver disease and respiratory diseases [169,170].

Moreover, long COVID sequelae have also become apparent in individuals with asymptomatic, mild and moderate COVID-19 [171,172]. Before acquiring the disease, many of these individuals reported a healthy state without underlying conditions. A multi-centre study found that within these low risk individuals, there was evidence of mild organ impairment, particularly in the heart (32%) and lungs (33%) [171]. While evidence of long COVID in adults has been systematically reviewed, details of paediatric cases are beginning to emerge. Findings suggest that children may experience similar long COVID symptoms to adults and female children were more at risk than males of the same age [172]. As such, the level of organ damage experienced by younger healthy adults and children with long COVID is a major concern, since the development of long COVID could greatly impact future quality of life by predisposing otherwise healthy people to chronic ailments. The challenge therefore lies in determining the mechanisms responsible for the development of long COVID, so that pharmacological intervention can be established. This is particularly important in cases where the vaccine has not yet been administered or is not effective. As in general, COVID-19 vaccines have the potential to attenuate the severity of infection and also reduce transmission [22]. In particular, therapeutics that target aspects of the RAAS and the KKS described above may present as a viable option for the attenuation of long COVID symptoms.

## 8. Therapeutic Potential

Unsurprisingly, the plethora of signalling pathways and physiological responses associated with RAAS and KKS dysregulation has led to a number of pharmacological agents effective in treating cardiovascular disease (Table 1). Drugs that target the AT_1_R (ARBs and ACEis) are successfully used to attenuate symptoms of hypertension by reducing the pathogenic actions of Ang II. In addition, biased AT_1_R ligands that signal via β-arrestin or G_αi_ have shown promise in alleviating the adverse cardiovascular effects associated with RAAS dysfunction [57,173]. These ligands may be an interesting avenue to explore for prospective COVID-19 treatments. Additionally, it has been proposed that stimulation of AT_2_Rs could be an effective approach to counteract the imbalance of RAAS during COVID-19 [174]. A small molecule AT_2_R agonist, C21, has been shown to reduce fibrosis, hypertrophy and the release of pro-inflammatory cytokines; as well as improving heart function [175]. In addition, it has been suggested that C21 may reduce prolonged pulmonary dysfunction in COVID-19. Targeting AT_2_Rs to alleviate severe pulmonary symptoms and CVCs of COVID-19 may be an effective strategy [176]. Particularly as C21 is currently undergoing phase 2 clinical trials for the safety and efficacy evaluation in patients with COVID-19. Further understanding of AT_2_R signalling could lead to improvement of AT_2_R drug development which may have future implications for COVID-19 CVCs, long COVID and cardiovascular diseases.

Furthermore, ACE2 and MasR agonists have shown promise in counterbalancing aberrant RAAS signalling. For example, xanthenone, an ACE2 activator, reduces BP in spontaneously hypertensive rats; attenuated myocardial, renal and pulmonary fibrosis and stimulated vascular repair [177,178,179]. Whereas the MasR agonist CGEN-856S effectively induces vasorelaxation, improves endothelial function and induces cardio-protection [180]. Although these compounds show potential for the treatment of hypertension, cardiovascular diseases and COVID-19 CVCs, they have demonstrated a lack of efficacy in different preclinical models due to their solubility or rapid metabolism and low bioavailability in vivo [133]. To overcome these issues, the hydrophobicity and stability of ACE2 and MasR agonists needs to be considered during the drug development process.

Similar to the RAAS, small molecules that target components of the KKS have been hypothesised as useful agents for the treatment of both respiratory and cardiovascular symptoms of COVID-19 [77]. For example, a small cohort study demonstrated that the B_2_ receptor antagonist icatibant did not improve the mortality of severe COVID-19 patients [181]. However, it did promote significant improvements in lung health and eosinophil blood counts, which are indicative of clinical improvement [181]. This KKS antagonistic approach for COVID-19 treatment may highlight a potential avenue for drug development. However, currently, few drugs that target kinin receptors have been approved for clinical use and only a limited number of studies have begun to investigate kinin receptors in COVID-19. Therefore, a significant amount of research into the KKS and COVID-19 needs to be conducted so that clear conclusions can be drawn.

## 9. Conclusions

Here, we have reviewed the potential impact of SARS-CoV-2 on the RAAS and KKS as a consequence of the virus using ACE2-mediated endocytosis as a viral entry mechanism, leading to a loss of this enzyme from the cell surface. ACE2 has an important role in regulating the metabolism of both Ang II and DABK and the subsequent formation of angiotensin and bradykinin metabolites. It is therefore not surprising that the severity of COVID-19 has been linked to cardiovascular co-morbidities and that long COVID manifests itself with many CVCs. As a consequence, therapeutic targets have been identified within the RAAS and KKS that could potentially offer novel approaches to the prevention and management of CVCs associated with COVID-19. The use of AT_1_R antagonists and ACEi is the current mainstay of the treatment of hypertension and associated disorders, and there is evidence that the use of ACEi and ARBs, despite initial concerns, can re-establish RAAS homeostasis in severe COVID-19. Unfortunately, there is limited information available for the role of AT_2_R, MasR and ACE2-directed molecules and further research is required. This is also true for drugs targeted at the KKS, although a B_2_ receptor antagonist and monoclonal antibody that inhibits plasm kallikrein activity is in clinical use for the treatment of hereditary angio-oedema. It is clear, therefore, that despite the extensive history of research into both the RAAS and KKS, there is still more to do from the context of ACE2 which has been, and is likely to continues to be, targeted by existing and new coronaviruses.

## Figures and Tables

**Table 1:** G-protein coupling associated with the receptors found within the RAAS and KKS.

Receptor	G Protein Signalling	Cellular Actions	Physiological Response
AT_1_R	G_αq_	DAG, PKC, ↑NO, ↑Ca^2+^, NHE3 activation	Vascular constriction, renal sodium retention (↑H^+^ secretion, ↑Na^+^ absorption), ↑ROS
	G_αi2_ G_αi3_	↓cAMP, activates GIRKs	↑parasympathetic pathways, ↓HR, ↓BP
	G_α12_ βarrestin2	Rho GTPase, TKs, NADPH oxidases Receptor desensitisation, internalisation	↑actin stress fibres, ↑focal adhesions, ↑cell growth, ↑fibrosis, ↑hypertrophy Dampens AT_1_ physiological effects
AT_2_R	G_αs_ G_ai/0_ G_αi2_ G_αi3_ Non-canonical	↑cAMP ↑eNOS ↓TKs ↑BK/cGMP/NO	Muscle repair, vasorelaxation, ↑paracrine signalling
		↓IP3, ↑NOS, ↓Na^+^ATPase, ↓PLD ↓Rho	Inhibition of AT1 responses
MasR	Constitutively activates G proteins G_αq_ G_αi_ βarrestin2	Exact role of ligand mediated G protein coupling not yet known.	NO-dependant vasorelaxation, protects endothelial function, ↓thrombosis ↓inflammation No measurable effect of Ca^2+^
		Receptor Internalisation, ↑ERK1/2, AKT, PLA2 ↓cAMP	Attenuates Ang((1-7)-)mediated activity at
B_1_ receptor	G_αq_ G_αi_	PLC, AKT, iNOS, ↑NO, ↑Ca^2+^ ↓cAMP, activates GIRKs, PLA	Vasorelaxation Release of arachidonic acid and prostaglandins Sustained activation-long term inflammation
B_2_ receptor	G_αq_ G_αi_ βarrestin	PLC, AKT, iNOS, ↑NO, ↑Ca^2+^ ↓cAMP, activates GIRKs, PLA Internalisation/receptor recycling	Vasorelaxation Release of arachidonic acid and prostaglandins Desensitisation- short term effects

G_aq_, G_ai2_, G_ai3_, G_ai/0_, G_a12_, and βarrestin have been found to interact with AT_1_R, AT_2_R, MasR, and bradykinin receptors (B_1_R and B_2_R) at varying intensities. These couplings result in distinct cellular signalling and physiological responses [39,53,54,55,56,57,58,59].

## Abbreviations

ACE2	angiotensin converting enzyme 2
ACEi	angiotensin converting enzyme inhibitor
Ang II	angiotensin II
AP	aminopeptidases
ARB	angiotensin receptor blockers
ARDS	acute respiratory distress syndrome
ASA	aldosterone synthase antagonist
AT_1_R/AT_2_R	angiotensin type 1/2 receptor
B_1_R/B_2_R	bradykinin type 1/2 receptor
BP	blood pressure
BK	bradykinin
COVID-19	coronavirus disease 2019
CVCs	cardiovascular complications
CPM	carboxypeptidase M
DABK	des-Arg^9^-bradykinin
DAKD	des-Arg^10^-kallidin
DPPD-4	dipeptidyl peptidase 4
GPCRs	G protein-coupled receptors
ICU	intensive care unit
IFN	interferon
IL-6	interleukin-6
KD	kallidin
KKS	kinin–kallikrein system
NETs	neutrophil extracellular traps
NLRP3	NOD-, LRR- and pyrin domain-containing protein 3
RAAS	renin–angiotensin–aldosterone system
ROS	reactive oxygen species
SARS-CoV-2	severe acute respiratory syndrome coronavirus 2
TMPRSS2	transmembrane protease serine 2
TNFα	tumour necrosis factor α
